# Self-rated health and related drivers that influences on health: A cross-sectional study among adults living in Riyadh, Saudi Arabia

**DOI:** 10.1097/MD.0000000000045268

**Published:** 2025-10-17

**Authors:** Abdullah M. Alobaid, Naji Alqahtani, Adel Bashatah, Kholoud Alharbi, Wajid Syed

**Affiliations:** a Department of Accidents and Trauma, Prince Sultan Bin Abdulaziz College for Emergency Medical Services, King Saud University, Riyadh, Saudi Arabia; b Department of Nursing Administration and Education, College of Nursing, King Saud University, Riyadh, Saudi Arabia; c Department of Clinical Pharmacy, College of Pharmacy, King Saud University, Riyadh, Saudi Arabia.

**Keywords:** depression, physical activity, Saudi Arabia, stress, workplace

## Abstract

An individual’s health influences the socioeconomic burden at both individual and organizational levels. Therefore, this study aimed to assess self-rated health and its factors that influence health among adults living in Riyadh, Saudi Arabia. A cross-sectional study was conducted to assess the self-rated health of Saudi adults and the factors that affect their health status. Data collection for this study took place between October and November 2024 using predefined self-administered questionnaires consisting of 4 sections with a total of 17 items. Data analysis was performed using the Statistical Package for Social Science version 27 (SPSS Inc., Armonk). The Chi-square or Fisher exact test was utilized to assess group differences, with *P* < .05 considered statistically significant. A response rate of 97.4% was obtained, with 64.5% of respondents being male. The majority of participants (83.4%) were aged between 20 to 30 years, and 88.5% held a bachelor’s degree. Most respondents (96.3%) rated their health status as good. However, 16.4% reported experiencing severe to moderate occupation-related stress, and 16.2% revealed depression. Notably, 85.4% of participants did not report occupation-related pain. The results revealed that occupation-related depression levels were significantly associated with educational qualifications (*P *< .001). Individuals with a graduate degree reported higher levels of severe occupation-related depression compared to those with other educational backgrounds. Moreover, individuals working in the private sector reported the highest frequency of moderate occupation-related depression compared to those employed in the government sector (*P* = .023). With regard to occupation-related pain, it was more prevalent among young adults aged 20 to 30 years compared to other age groups (*P* = .019). Similarly, educational qualifications (*P* < .001) and job sector (*P* = .004) were significantly associated with occupation-related pain. Finally, self-assessed health was found to be significantly associated with age (*P *= .020), educational qualifications (*P* = .002), and job sector (*P* = .047). The study concluded that the majority of adults were healthy, although some suffered from occupation-related stress and depression. Factors such as age and education were associated with self-rated health. To mitigate the negative health effects on adults’ quality of life and in organizations, appropriate surveillance, early recognition, treatment, addressing factors causing poor health and implementing occupation-related stress and depression management techniques are necessary.

## 1. Introduction

More generally, health is defined as a state of complete physical, mental, and social well-being rather than simply the absence of illness or disability.^[1,2]^ To lead a healthy lifestyle, health is a fundamental concept. An individual’s perception of their health is determined by their health status.^[3]^ Conversely, adults’ self-rated health serves as a subjective indicator they use to assess their well-being.^[4]^ Health measurement is crucial as it provides insight into how individuals perceive their health, which can differ from clinical assessments.^[1,3,5]^ It is commonly utilized as a screening tool in health surveys or to identify those at risk of developing an illness.^[1,3,5]^ According to Science Daily, over 95% of people worldwide experience health issues, with more than a 3rd dealing with 5 or more health conditions,^[6]^ the prevalence of chronic conditions is increasing; for instance, in 2022, over 1 billion individuals globally were obese, and 43% of adults were overweight.^[7]^ A recent review of 42 studies found that the global prevalence of depression was 19.2%, anxiety was 16.5%, and stress was 13.9%.^[8]^

In this modern world, the use of technology such as the internet and smartphones is at peak levels, with individuals busy browsing the internet and social media.^[9–11]^ Furthermore, most employment involves constant sitting, either in front of the computer or engaging in physically demanding work. This increased sitting and physical strain may have negative effects on health. For example, continuous or excessive screen time has led to unhealthy habits like prolonged sitting or sedentary behavior, which can result in the development of chronic diseases.^[12–14]^ A recent study among Swedish employees revealed that sitting almost all the time at work and not taking breaks is associated with an increased risk for self-reported poor general health and back/neck pain.^[14]^ Additionally, the literature suggests a high prevalence of sedentary behavior in Saudi Arabia (SA).^[10,11,15]^ Studies show that physical inactivity and sedentary behavior are major contributing factors to diabetes, cardiovascular disease, and obesity. Additionally, inactivity has been significantly correlated with sitting time and occupation status among adults in SA.^[11]^

Several reports from around the world have been published to explore the health status and related factors that influence health among public.^[14,16,17]^ According to literature, health is impacted by chronic conditions, mental health issues (stress, anxiety, and depression), lifestyle habits (smoking, drinking, and unhealthy diet), and social determinants (access to healthcare, education, and income level).^[17]^ A recent study found that self-rated poor health was significantly associated with lack of exercise, stress, chronic diseases, and occupation-related pain.^[16]^ In SA, education, marital status, and risky behaviors have been linked to health status.^[18]^ Research also suggests that work status is associated with psychological diseases.^[12,16,18]^ For example, individuals with jobs involving prolonged sitting or physically demanding tasks are advised to take regular breaks.^[14,16]^ Understanding health status and related factors is crucial for enhancing quality of life and preventing poor health among adults. However, previous studies have primarily focused on older and unemployed adults with chronic diseases,^[5,11,18]^ leaving a knowledge gap in understanding self-rated health among young employed adults in SA. This study addresses this gap by targeting young adults, a significant portion of Riyadh population, who are likely in the early stages of their careers. By examining the impact of occupation-related factors, lifestyle habits, and sociodemographic characteristics on their health, this study can provide valuable insights into the health needs of this population. As the future of the country, the health status of young adults significantly influences national development and economy. Therefore, understanding their health status and related factors can inform local health policies and interventions that promote healthy behaviors, reduce the risk of chronic diseases, and contribute to effective health strategies. By providing context-specific insights, our study aims to inform local health policies and interventions, ultimately enhancing the health and well-being of young employed adults in SA. This study aims to assess self-rated health and factors that influence health among adults living in Riyadh, SA.

## 2. Methods

### 2.1. Study design, population, and setting

A cross-sectional web-based online study was conducted to assess the self-rated health of Saudi adults and the factors and age-related drivers that affect their health status. Data collection for this study took place between October and November 2024, immediately after receiving ethical approval from the Human Ethics Committee for human research at King Saud University in Riyadh, SA. Additionally, Declaration of Helsinki principles for the best clinical practices were followed throughout the investigation. Young adults living in SA who were above 20 years of age, currently employed, Saudi nationals, and able to provide informed consent by completing the questionnaires were included in the study. Students, individuals living outside the country, or those who were unemployed were excluded from the study. The questionnaire included a statement at the beginning, explaining the purpose and significance of the study, as well as ensuring the confidentiality of the provided data. Respondents were assured that their data would be used solely for research purposes and that confidentiality would be maintained throughout the study. They were also informed of their right to withdraw from the study at any point in time. By responding to the questionnaire, individuals were considered to have provided informed consent.

### 2.2. Sample size and sampling procedure

Similar to earlier studies^[19–22]^ the sample size was calculated using the Raosoft sample size calculator based on the total population of Riyadh (7821,000). With a 95% confidence interval, the estimated sample size was 385. To avoid sampling bias or incomplete responses and to strengthen the study, we approached 500 individuals.

### 2.3. Questionnaire design and data collection

The questionnaire used in this study was adapted from previously published studies and consisted of 4 sections with a total of 17 items.^[16]^ The first section gathered demographic details of the adults (4 items) such as age, gender, education, and living status. The second part collected information on occupation-related characteristics, including job-status (high, moderate, or low pay), job type (government or private), work shift and hours worked per week, night work, time taken to travel from home to work, with a total of 6 items. The 3rd section focused on the presence of chronic diseases, exercise, and smoking status (3 items). The final part of the study inquired about occupation-related stress (yes/no), pain (yes/no), depression (yes/no), and self-rated health (good or poor) among respondents (4 items). We selected a variety of variables to thoroughly evaluate the factors that influence health among young adults. Occupation-related variables like stress were included to identify potential stressors and demands that impact health and contribute to stress.^[23]^ Additionally, we considered chronic diseases due to their impact on overall health, including negative effects on sleep patterns, mental health, and quality of life. Lifestyle factors such as regular physical activity, smoking, and self-rated health were also assessed, as they can significantly influence sleep quality, mental health, physical health, and overall well-being.^[24]^ Furthermore, the presence of depression was also assessed, which not only affects work but also overall health.^[25]^ By examining these variables, helps to gain insight into potential underlying mental health issues and correlations between lifestyle factors, sleep quality, and overall well-being.

First, the questionnaire was originally prepared in English and then translated into Arabic to ensure a better understanding of the questions using forward and backward translation procedures. The language validity was then confirmed by retranslating the Arabic version back into English to ensure the original meaning of the questions was preserved (back translation). The authors, who are bilingual speakers of both English and Arabic, conducted the back translation. After formulating the questionnaires, they were subjected to expert review for content, flow, and clear understanding of the questions. Before conducting the study, a pilot study was undertaken among randomly selected individuals. The reliability of the questionnaires was determined using Cronbach alpha, which was found to be 0.79, suggesting that the questionnaires are valid and reliable for conducting the study.

For data collection, electronic questionnaires were prepared using Google Forms and distributed via social media applications. The invitation link was sent through WhatsApp groups and posted on various community groups on Telegram, Facebook, and Instagram. The link to the questionnaires began with an agreement to participate in the study and an explanation of the study’s aims and inclusion criteria. Those who agreed to proceed were redirected to the original questionnaires. To increase the sample size further and reach the maximum number of respondents, participants were asked to distribute the survey among their social networks. To prevent duplicate submissions of responses and ensure data integrity, we restricted Google Forms to allow only 1 response per individual.

### 2.4. Data analysis

Data analysis was conducted using the Statistical Package for Social Science version 27 (SPSS Inc., Armonk). Descriptive analysis was utilized to determine frequencies (n) and percentages (%), and the Chi-square or Fisher exact test was employed to evaluate group differences. Results with a *P*-value of < .05 were deemed statistically significant.

## 3. Results

The study achieved a response rate of 97.4% (n = 500). After excluding 13 participants (2.6%) due to noncompliance with inclusion criteria (n = 8), incomplete questionnaires (n = 5), the final sample consisted of 487 participants. The majority of participants were male (n = 314; 64.5%) and aged between 20 to 30 years (n = 427; 87.7%). Most participants held a bachelor’s degree (n = 431; 88.5%), while a smaller percentage were pursuing postgraduate studies (n = 18; 3.7%), held doctorates (n = 7; 1.4%), or had lower educational qualifications. Table 1 displays the distribution of sociodemographic characteristics of the respondents.

**Table 1:** Distribution of sociodemographic and job characteristics of the adults.

Characteristics	Frequency (%)
Sex	
Male	314 (64.5)
Female	173 (35.5)
Age	
20–25	406 (83.4)
26–30	21 (4.3)
31–35	40 (8.2)
36–40	11 (2.3)
45–50	9 (1.8)
Qualifications	
Bachelor’s degree	431 (88.5)
Postgraduate studies	18 (3.7)
Doctorate	7 (1.4)
Diploma	22 (4.5)
Secondary school	9 (1.8)
Living with family	
Yes	334 (68.6)
No	153 (31.4)

### 3.1. Occupational related characters

In terms of work related characters, 38.2% (n = 186) were in low-wage positions, 46.8% (n = 228) in moderate-wage positions, and 15.0% (n = 73) in high-wage positions, with the majority working in the government sector (n = 308; 63.2%). Approximately 85.6% (n = 417) worked regular daytime hours (8 am to 3:30 pm), while others had varied shifts, including night work (n = 52; 50.1%). In terms of working circumstances, the majority of participants worked 40 hours per week (n = 442; 90.8%), with some reporting longer hours. A small percentage (n = 61; 12.5%) worked at night for 1 to 5 days each month. Commute times were split, with 45.2% (n = 220) taking less than an hour and 54.8% (n = 267) requiring more than an hour to get to work (Table 2).

**Table 2:** Distribution of occupational related characters of the adults.

Variable	Frequency (%)
Job-status	
Low-paying job	186 (38.2)
Moderately paid job	228 (46.8)
A high-paying job	73 (15.0)
Job type*	
Government sector	308 (63.2)
Private sector	178 (36.6)
Work shift	
Daily work (8 am to 3.30 pm)	417 (85.6)
Night work	25 (50.1)
Afternoon (12 noon to 8 pm)	15 (3.1)
Mixed shifts	30 (6.2)
Working hours per week	
40 h	442 (90.8)
41–45 h	31 (6.4)
46–50 h	13 (2.7)
51–60 h	1 (.2)
Night work, monthly days	
1–5 d	63 (12.9)
6–10 d	19 (3.9)
>11 d	2 (.4)
More than 11 d	18 (3.7)
No night shift	387 (79.5)
How long does it take to get from your home to work?	
Less than an hour	220 (45.2)
More than an hour	267 (54.8)

* Missing response.

### 3.2. Status of exercise and chronic disease

**Figure 1.**
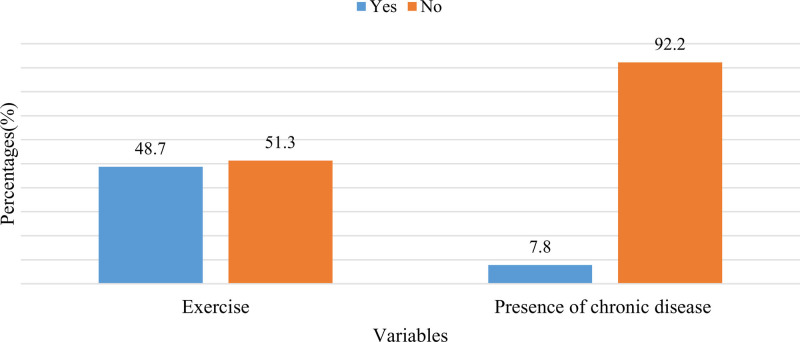
Status of exercise and chronic disease.

Exercise habits varied among the respondents, with 48.7% (n = 237) reporting that they exercised, while 51.3% (n = 250) did not. Chronic diseases were prevalent among 7.8% of the respondents (n = 38). For a detailed breakdown of respondent frequencies, please refer to Figure 1.

### 3.3. Frequency of sleeping pattern and smoking status

Regarding sleep patterns, 39.4% (n = 192) of participants reported sleeping 7 to 8 hours per day, while 23.2% (n = 113) slept <6 hours. Additionally, lifestyle habits analysis revealed that 15% (n = 73) were current smokers, and 40.9% (n = 199) engaged in physical activity for 30 minutes or less per week (see Table 3 for details).

**Table 3:** Distribution of sleeping pattern and smoking status among respondents.

Variable	Frequency (%)
Smoking status	
Currently smoking	73 (15.0)
Former smoker	29 (6.0)
I don’t smoke	385 (79.1)
Number of hours of sleep per day	
<6 h	113 (23.2)
6–7 h	162 (33.3)
7–8 h	192 (39.4)
More than 8 h	20 (4.1)

### 3.4. Frequency of occupation-related stress, depression, and self-rate health

Table 4 presents findings on occupation-related stress, depression, and self-rated health. Notably, 16.4% of respondents (n = 80) experienced moderate to severe stress, whereas 68% (n = 331) reported no stress. Occupation-related depression levels varied, with 10.1% (n = 49) reporting low levels, 83.8% (n = 408) indicating no depression, and 1.0% (n = 5) experiencing severe depression. Furthermore, 85.6% of participants (n = 417) did not report occupation-related pain, and the majority rated their health as good.

**Table 4:** Distribution of occupation-related stress, depression, and self-rate health.

Variable	Frequency (%)
The presence of stress	
High	25 (5.1)
Moderate	55 (11.3)
A little	76 (15.6)
There is no pressure	331 (68.0)
Do you feel depressed because of your work (work/travel)?	
Severe depression	5 (1.0)
In moderation	25 (5.1)
A little	49 (10.1)
I do not suffer from depression	408 (83.8)
Occupation related pain	
Yes, I suffer from work pain	71 (14.6)
No, I don’t feel pain	416 (85.4)
Self-assessment of health	
My health condition is bad	18 (3.7)
My health condition is not bad	469 (96.3)

### 3.5. Associations between occupation-related depressions and pain

To find out the associations between occupation-related depression, work related pain, and various factors, including age, gender, qualifications, job, living status, and job type, Chi-square and Fisher exact tests (*P *< .05) was carried out. The results revealed significant associations between depression levels and qualifications were significantly associated with depression levels (*P* < .001), with individuals with graduation reporting higher levels of severe depression compared to other educations. On the other hand individuals working in private sector reported highest frequency of moderate depression, comparing to individuals working in government sector. Similarly, majority of the individuals working in government sector did not reported depression. Suggesting that job type has significantly associated occupation-related depression among respondents (*P* = .023). However, gender, job, living with and family, were not significantly associated with depression levels. Regarding occupation-related pain it was higher among young adults aged between 20 to 30 years comparing to other age category, suggesting that age (*P* = .019) was significantly associated with work related pain. Similarly, individual’s qualifications (*P* < .001), and job sector (*P* = .004) were significantly associated, while gender, job type, and living situation were not (Table 5).

**Table 5:** Association between occupations related depression, pain and demographics.

Variables		Occupation-related depression				*P*	Occupation-related pain		*P*
		Severe	Moderate	little	Don’t suffer		Yes	No	
Gender						5.763 Df = 3 .120			2.507 Df = 1 .072
Male	Count	3	17	39	255		51	263	
	% within sex	1.0%	5.4%	12.4%	81.2%		16.2%	83.8%	
Female	Count	2	8	10	153		19	154	
	% within sex	1.2%	4.6%	5.8%	88.4%		11.0%	89.0%	
Age						14.985 Df = 12 .254			12.046 Df = 4 .019
20–25	Count	4	19	38	345		54	352	
	% within age	1.0%	4.7%	9.4%	85.0%		13.3%	86.7%	
26–30	Count	1	3	2	15		4	17	
	% within age	4.8%	14.3%	9.5%	71.4%		19.0%	81.0%	
31–35	Count	0	3	8	29		12	28	
	% within age	0.0%	7.5%	20%	72.5%		30.0%	70.0%	
36–40	Count	0	0	1	10		0	11	
	% within age	0.0%	0%	9.1%	90.9%		0%	100%	
45–50	Count	0	0	0	9		0	9	
	% within age	0%	0%	0%	100%		0%	100%	
Qualifications						65.503 Df = 12 <.001			19.138 Df = 4 <.001
Bachelor’s	Count	3	18	45	365		57	374	
	% within Qualification	0.7%	4.2%	10.4%	84.7%		13.2%	86.8%	
Postgraduate	Count	0	0	1	17		2	16	
	% within Qualification	0%	0%	5.6%	94.4%		11.1%	88.9%	
Doctorate	Count	0	0	0	7		0	7	
	% within Qualification	0%	0%	0%	100%		0.0%	100.0%	
Diploma	Count	0	5	3	14		10	12	
	% within Qualification	0%	22.7%	13.6%	63.6%		45.5%	54.5%	
Secondary school	Count	2	2	0	5		1	8	
	% within Qualification	22.2%	22.2%	0%	55.6%		11.1%	88.9%	
Job type						3.698 Df = 6 .730			2.427 Df = 2 .308
Low-paying	Count	3	12	20	151		32	154	
	% within job	1.6%	6.5%	10.8%	81.2%		17.2%	82.8%	
Moderately paying	Count	1	10	20	197		27	201	
	% within job	0.4%	4.4%	8.8%	86.4%		11.8%	88.2%	
High-paying	Count	1	25	49	408		11	62	
	% within job	1.0%	5.1%	10.1%	83.8%		15.1%	84.9%	
Living with family						2.347 Df = 3 .527			2.795 Df = 1 .064
Yes	Count	5	17	34	278		42	292	
	% within living with family	1.5%	5.1%	10.2%	83.2%		12.6%	87.4%	
No	Count	0	8	15	130		28	125	
	% within living with family	0%	5.2%	9.8%	85.0%		18.3%	81.7%	
Job type						22.702 Df = 6 .023			11.175 Df = 2 .004
Government	Count	3	5	29	271		32	276	
	% within a job type	1.0%	1.6%	9.4%	88.0%		10.4%	89.6%	
Private	Count	2	20	20	136		38	140	
	% within a job type	1.1%	11.2%	11.2%	76.4%		21.3%	78.7%	

### 3.6. Associations between self-rated health and demographics

The results of the associations between self-assessed health and demographics, including gender, age, qualifications, job type, income, living situation, and job sector, using Chi-square tests (Table 6). Significant associations were found between self-assessed health and age (*P* = .020) and qualifications (*P* = .002), and job sector (*P* = .047) while gender, living situation, showed no significant associations.

**Table 6:** Association between self-rated health and demographics.

Variables		Self-rated health		*P*-value
		Good health	Bad health	
Gender				2.902 Df = 1 .068
Male	Count	299	15	
	% within sex	95.2%	4.8%	
Female	Count	170	3	
	% within sex	98.3%	1.7%	
Age				15.111 Df = 4 .020
20–25	Count	393	13	
	% within age	96.8%	3.2%	
26–30	Count	17	4	
	% within age	81.0%	19.0%	
31–35	Count	39	1	
	% within age	97.5%	2.5%	
36–40	Count	11	0	
	% within age	100.0%	0%	
45–50	Count	9	0	
	% within age	100.0%	0%	
Qualifications				30.282 Df = 4 .002
Bachelor’s	Count	419	12	
	% within Qualification	97.2%	2.8%	
Postgraduate	Count	18	0	
	% within Qualification	100%	0	
Doctorate	Count	7	0	
	% within Qualification	100%	0	
Diploma	Count	19	3	
	% within Qualification	86.4%	13.6%	
Secondary school	Count	6	3	
	% within Qualification	66.7%	33.3%	
Job				3.298 Df = 2 .217
Low-paying job	Count	178	8	
	% within job	95.7%	4.3%	
Moderately paid job	Count	218	10	
	% within job	95.6%	4.4%	
High-paying job	Count	73	0	
	% within job	100%	0%	
Living with family				0.733 Df = 1 .282
Yes	Count	320	14	
	% within living with family	95.8%	4.2%	
No	Count	149	4	
	% within living with family	97.4%	2.6%	
Job type				7.321 Df = 2 .047
Government sector	Count	6	302	
	% within a job type	1.9%	98.1%	
Private sector	Count	12	166	
	% within a job type	6.7%	93.3%	

## 4. Discussion

Despite the numerous efforts of individuals, healthcare organizations, and professionals, maintaining health and wellness remains challenging, especially among working professionals and adults worldwide. This challenge is driven by various factors such as occupation-related stress, social pressures, economic constraints, and cultural influences. This study revealed that majority of individuals reported good health, while 3.7% reported poor health. However, occupation-related issues like occupation-related pressure (16.4%) and occupation-related pain (14.6%) were reported among participants. These findings were consistent with earlier studies conducted in other countries.^[26,27]^ For instance, a study in Australia from 2017 to 2018 found that 56.4% of Australians rated their health as good, while only 3.7% rated it as poor.^[27]^ Similarly, research from a developing country with a large sample size of 46,483 individuals showed that 9.42% of them had poor self-rated health.^[26]^ Therefore, individuals’ perceptions of their health often serve as a proxy for their actual health status, and their experiences with illness and disability greatly influence their self-assessment of health. This measure is therefore a crucial indicator of key aspects of quality of life.^[27]^

Concerning lifestyle habits, 48.7% of the adults were exercising and 39.4% of them were sleeping 7 to 8 hours, while only 15% of participants were current smokers, and 40.9% of them engaged in physical activity for 30 minutes or less per week. In addition, 7.8% of them reported having chronic diseases, which might be the reason for the self-reported good health among respondents. However, earlier studies show similar findings. For example, a study by Bashatah et al among young adults revealed that 63% of the adults had physical activity every week. Walking (45.2%) followed by bodybuilding (12.7%) was the most common type of physical activity identified among adults in SA.^[10]^ Similarly, another study in SA revealed that 44% of the respondents exercised 1 to 2 days a week, and 16.7% never exercised. Furthermore, a considerable percentage of the respondents spent more than 4 hours a day being sedentary, and most of the sedentary time was spent on occupation-related activities (62%), followed by time spent on coffee (36.4%), and business-related activities (22.5%).^[28]^ These findings suggest that constant sitting at the workplace exists and might have effects on the overall health of individuals.

In this study, 16.2% of the individuals were found to be suffering from occupation-related depression. Furthermore, the prevalence of depression was higher among young adults aged between 20 to 30 years compared to others. These findings were consistent with earlier national and international research. For example, a previous study showed that 18.6% of adults aged 18 to 25 were experiencing depression.^[29]^ Similarly, a study among working professionals found that 19.1% of them were dealing with occupation-related depression, with 36.3% experiencing moderate to severe levels of depression.^[30]^ Additionally, another study found that between 10% to 52.9% of working individuals were suffering from depression.^[31]^ A study focused on assessing the mental health and well-being of employees found that depression was the most common issue, affecting 59% of workers.^[32]^ Previous reports have suggested that adults aged 18 to 25 are more likely to experience at least 1 major depressive episode.^[29,33]^ In the United States, a study conducted in 2020 revealed that approximately 1 in 5 adults reported having received a diagnosis of depression at some point in their lives, with prevalence being higher in women, younger adults, and those with lower education levels.^[29,34]^

In the current study, 16.4% of participants reported experiencing severe to moderate occupation-related stress. Stress in the workplace is common, as evidenced by numerous studies conducted both nationally and internationally. For example, a study by Aziz et al in Jeddah found that 52.7% of working professionals in SA experienced occupation-related stress.^[30]^ Another study reported that occupation-related stress levels ranged from 3.8% to 75.5%.^[31]^ A study of a large population (n = 3995) concluded that 47% of employees suffered from occupation-related stress, followed closely by financial stress at 46%.^[32]^ In Canada, recent literature showed that 4.1 million people reported high or very high levels of occupation-related stress, accounting for 21.2% of all working individuals.^[35]^ Given these findings, it is important to identify the factors contributing to occupation-related stress and depression. Previous research has suggested that factors such as heavy workloads, balancing work and personal life, and being female can lead to high levels of occupation-related stress.^[35]^

Similarly, the prevalence of occupation-related depression was significantly higher among adults with a bachelor’s degree compared to those with other educational qualifications. Additionally, severe depression was more common among individuals in high-paying jobs, followed by those in moderately paying jobs, as opposed to those in low-paying jobs. This suggests that occupation-related depression is significantly associated with job type. Previous research has shown that depression, in general, is more prevalent among females, single individuals, and undergraduate university students in university settings. Risk factors significantly associated with depression include female gender, being single, a low level of education, financial problems, poor housing conditions, medical problems, sleep disorders, psychiatric/psychological conditions, life events, lack of social support, exposure to stress, educational/personal problems, and smartphone addiction.^[36]^

In this study, occupation-related pain was found to be higher among young adults, diploma holders, and those working in the private sector. This suggests that age, education, and employment sectors are significantly associated with occupation-related pain among Saudi adults. A previous study by Pooleri et al among young adults revealed that between 10% and 3% experience pain interference at a young age.^[37]^ Due to work pain, 43% of respondents in the previous study had exited the labor force at least once, and 10% developed a new occupation-related health limitation.^[37]^ Therefore, workplace-related diseases and health status need to be recognized early, and providing immediate solutions and treatment may help young workers reduce the burdens of future unemployment and disability.

The findings showed that self-rated health among adults was significantly associated with age and educational qualifications. Gender, however, was not significantly associated with self-rated health among adults. These findings were comparable to earlier research^[38]^ where the author reported that the prevalence of any occupation-related health problem did not vary significantly by gender, but there was significant variation by age group and education, which aligns with the current findings.^[38]^ For instance, 21.7% of respondents aged 18 to 24 years reported poor health, while adults aged above 45 years were found to have the highest prevalence of poor health.^[38]^ Similarly, by educational attainment, poor health was highest (39.2%) among respondents with less than a high school diploma and lowest (30.6%) among those with a bachelor’s degree or higher.^[38]^ Another study among Australians revealed that older adults generally rated themselves as having poorer health than younger adults.^[4]^ There was little difference in the way men and women assessed their overall health, with men slightly more likely to report their health as fair or poor.^[4]^ Self-assessed health status is frequently used as a stand-in for actual health status, and people’s perceptions of their health are closely linked to their experiences with illness and impairment. Thus, this metric serves as a significant gauge of critical facets of life quality.

While occupation-related stress, depression, and pain may contribute to poorer health outcomes, as reported in earlier studies,^[39]^ good health is intricately linked to various social determinants.^[40]^ According to the conceptual framework for social determinants of health, complex relationships exist between employment, working conditions, transportation, and factors like occupation-related stress and depression ultimately influencing health outcomes and exacerbating health inequities.^[40]^ Poor working conditions, including nonstandard work hours and high job demands, can lead to various health issues, such as depression and anxiety, negatively impacting overall well-being. Conversely, good working conditions characterized by a safe environment, job security, and effective management can improve health outcomes and enhance well-being.

This study has several limitations firstly the cross-sectional nature of the study designs limits the ability to establish causality between variables. Secondly the use of self-administered questionnaires may introduce bias due to subjective reporting and potential misclassification of health status. Thirdly the study’s reliance on social media recruitment and the nonrandom online sample which may have introduced selection bias, potentially over representing young, educated, and digitally connected individuals. This limitation may affect the generalizability of the findings to the broader population of young employed adults in SA. Future studies should consider using mixed-method sampling strategies to reach a more diverse population. Furthermore, the multiple subgroup analyses in this study may increase the risk of Type I error inflation. Future research should take this limitation into consideration and possibly implement corrections for multiple comparisons, such as the Bonferroni correction, or use alternative statistical approaches to reduce this risk.

This study suggests that employers can take several steps to support employee health and well-being. Implementing stress management programs, such as mindfulness or cognitive-behavioral therapy, and encouraging physical activity through workplace wellness programs can help reduce stress and the risk of chronic diseases. Flexible work arrangements, like telecommuting or flexible hours, can also improve work-life balance and overall well-being. Additionally, providing targeted support for employees with lower qualifications can help address higher levels of severe depression. Future research should focus on longitudinal studies to examine the long-term effects of occupation-related stress, depression, and pain, and evaluate interventions aimed at promoting employee health.

## 5. Conclusion

The study revealed that while most adults were healthy, some experienced occupation-related stress and depression, with age and education being associated factors. To mitigate these negative health effects, workplace mental health initiatives and support systems are essential. This can be achieved by identifying high-risk groups, such as younger adults and those with lower qualifications, and providing targeted interventions. Establishing surveillance systems to monitor occupation-related stress and depression can also inform evidence-based interventions. By prioritizing workplace mental health, employers and policymakers can promote employee well-being and improve public health.

## Acknowledgment

The authors of this study extend their appreciation to the Ongoing Research Funding Program (ORF-2025-1099), King Saud University, Riyadh 11451, Saudi Arabia.

## Author contributions

**Conceptualization:** Abdullah M. Alobaid, Adel Bashatah, Wajid Syed.

**Data curation:** Abdullah M. Alobaid, Naji Alqahtani, Adel Bashatah, Wajid Syed.

**Formal analysis:** Abdullah M. Alobaid, Naji Alqahtani, Adel Bashatah, Kholoud Alharbi, Wajid Syed.

**Funding acquisition:** Abdullah M. Alobaid, Naji Alqahtani, Adel Bashatah, Kholoud Alharbi, Wajid Syed.

**Investigation:** Naji Alqahtani, Wajid Syed.

**Methodology:** Naji Alqahtani, Adel Bashatah, Kholoud Alharbi, Wajid Syed.

**Project administration:** Naji Alqahtani, Adel Bashatah, Kholoud Alharbi, Wajid Syed.

**Resources:** Abdullah M. Alobaid, Wajid Syed.

**Software:** Wajid Syed.

**Supervision:** Adel Bashatah, Wajid Syed.

**Validation:** Abdullah M. Alobaid, Kholoud Alharbi, Wajid Syed.

**Visualization:** Adel Bashatah, Kholoud Alharbi, Wajid Syed.

**Writing – original draft:** Abdullah M. Alobaid, Naji Alqahtani, Adel Bashatah, Kholoud Alharbi, Wajid Syed.

**Writing – review & editing:** Abdullah M. Alobaid, Naji Alqahtani, Kholoud Alharbi, Wajid Syed.
